# Design and Implementation of Trace Inspection System Based upon Hyperspectral Imaging Technology

**DOI:** 10.1155/2022/9524190

**Published:** 2022-07-15

**Authors:** Yuchen Wang, Zhongyuan Ji

**Affiliations:** ^1^College of Criminal Justice, Shandong University of Political Science and Law, Jinan 250014, China; ^2^Key Laboratory of Evidence-Identifying in Universities of Shangdong, Shandong University of Political Science and Law, Jinan 250014, China; ^3^College of Electronic and Information Engineering, Nanjing University of Aeronautics and Astronautics, Nanjing 211106, Jiangsu, China

## Abstract

Trace inspection is a key technology for collecting crime scenes in the criminal investigation department. A lot of information can be obtained by restoring and analyzing the remaining traces on the scene. However, with the development of digital technology, digital trace inspection has become more and more popular. So, the main research of this article is the design and realization of the trace inspection system based on hyperspectral imaging technology. This article proposes nondestructive testing technology in hyperspectral imaging technology. Combining basic principles of spectroscopy and the image of residual traces such as car tires, shoe soles, and blood stains, it can identify the key traces. Then, based on the image denoising and least squares support vector machine method, this study improves the accuracy and restoration of the image. Therefore, this study designs a test for the trace inspection system for testing hyperspectral imaging technology. The test items include the performance of the trace inspection system, the noise reduction of the trace inspection system, and the ability of the trace inspection system to inspect blood stains. The final collected data are improved to get the trace inspection system based on hyperspectral imaging technology proposed in this study. Compared with the traditional trace inspection system, the experimental results show that the trace inspection system based on hyperspectral imaging technology can improve the accuracy by 5%–28%, compared with the traditional trace inspection system. The image restoration degree of the hyperspectral imaging technology trace inspection system can be improved by 1%–19%, compared with the traditional trace inspection system.

## 1. Introduction

The universality of traces at the crime scene, material objectivity, close correlation with criminal behavior, and obvious intuition play an important role in criminal activities. Through the analysis and investigation of traces, we can judge the implementation process and specific circumstances of the crime and provide the direction and clues of the investigation. It provides a reliable basis for compound investigations and important physical evidence to prove facts. The last trace may be file storage to provide clues and evidence for investigating the current incident. Traditional trace inspection technology cannot adapt to the current complex and intelligent crime situation due to its low accuracy and inspection efficiency. Therefore, it is necessary to reform the past trace inspection technology.

With the maturity of hyperspectral imaging technology, it has been applied to many fields such as aerospace tongue coating imaging and pork detection. Its powerful functions are gradually brought into play and have attracted the attention of various countries. The research of tracking detection system based on hyperspectral imaging technology has a wide range of research space and application prospects. So, this study designs a trace inspection system based on hyperspectral imaging technology.

Saliency detection is a hot topic in recent years, and the results of saliency detection are difficult to use in general applications. Wang et al. believed that the main reason is the unclear definition of salient objects. In order to solve this problem, he claimed that the saliency should be defined in the context and took the saliency band selection in the hyperspectral image (HSI) as an example [1]. He studied the application of hyperspectral images in saliency detection, and this article mainly studies the application of hyperspectral images in trace inspection. Deep learning that represents data through hierarchical networks has been proven effective in computer vision. In order to study the role of depth features in hyperspectral image (HSI) classification, Ma et al. focused on how to extract and use depth features in the HSI classification framework [2]. Ma et al. studied the results of hyperspectral images in deep learning. If trace detection can be analyzed, it will be more in line with the purpose of this article. In order to study the application of hyperspectral images in SAJSRC, Fu et al. proposed a new shape-adaptive joint sparse representation classification (SAJSRC) method for hyperspectral image (HSI) classification. The method he proposed adaptively explores spatial information and incorporates it into a joint sparse representation classifier [3]. He is studying the application of hyperspectral images in adaptive joint sparse representation classification, and this article mainly focuses on trace inspection. The objective function of the classical non-negative matrix factorization (NMF) is nonconvex, which affects the obtaining of the optimal solution. Yan et al. proposed an NMF algorithm, which is based on the constraints of minimizing endmember spectral correlation and maximizing endmember spectral difference [4]. The method he proposed is based on the endmember spectrum, and this article studies hyperspectral images. Although there is little correlation, it still has a certain reference value. Shao et al. introduced an effective method for estimating the structure of probability classes. The SSL graph based on sparse representation adopts a method based on edge weighting, adding probability structure information to the sparse representation model. The graph construction method he proposed is superior to several traditional methods [5]. In September 1991, while hiking in the mountains of southern Austria, very close to northern Italy, Erika, and Helmut Simon stumbled upon the upper part of a human corpse protruding from a glacier. Larcher judged by the method of trace inspection that this was a hiker who disappeared in the area a few years ago [6]. He used the method of trace inspection to determine the identity of the corpse, which has a certain reference value for this article, but it is not great. In the oxygen minimum zone (OMZ), the oxygen concentration is at the limit of analytical detection. However, it does not undergo sulfate reduction, which is called hypoxia. Nitrate is usually used as the terminal electron acceptor for heterotrophic respiration. This respiration is highest near the top of the OMZ, where Cutter et al. observed the maximum of nitrite and other redox-active substances [7]. Its research is to detect the lowest area of oxygen, and this article mainly studies trace inspection. Hyperspectral images provide a wealth of spectral information for remotely distinguishing subtle differences in ground cover plants. The ever-increasing spectral dimensions and information redundancy make the analysis and interpretation of hyperspectral images a challenge. Zhao et al. proposed a new nonlinear feature extraction method for hyperspectral images [8]. The nonlinear feature extraction method of hyperspectral image proposed by Zhao et al. mainly studies feature extraction, while this article mainly studies trace inspection. Most of the documents cited in this article are about hyperspectral images, and trace inspection is rare, so this article needs to study the related knowledge of trace inspection in depth.

The innovation of this study is to combine the nondestructive testing technology with the hyperspectral imaging technology to analyze residual traces such as car tires, shoe soles, and blood stains. Through the method of image denoising and least square support vector machine, this study further refines and restores the trace image. Compared with the traditional trace inspection system based on hyperspectral imaging technology, the trace inspection system has the characteristics of high accuracy and high image recovery ability. This study also designed experiments to verify the performance of the trace inspection system, the noise reduction of the trace inspection system, and the ability of the trace inspection system to inspect blood stains.

## 2. Combined with the Application Method of Hyperspectral Imaging Technology in Trace Inspection

### 2.1. Hyperspectral Image Acquisition Method

**Figure 1:**
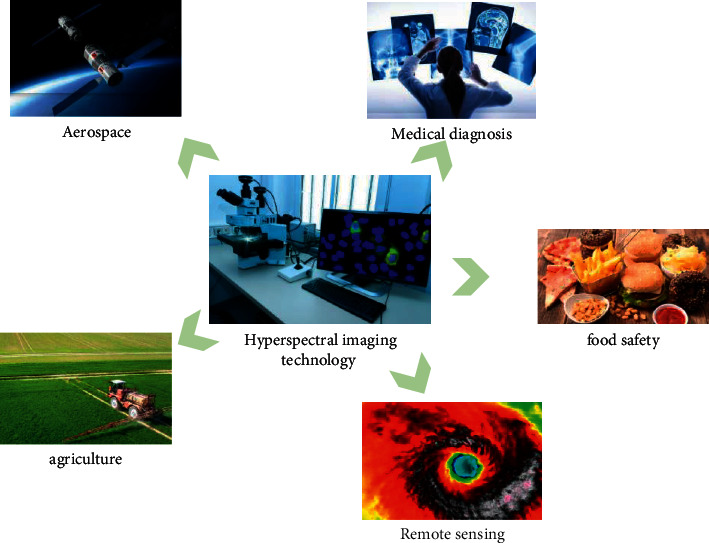
Application of hyperspectral imaging technology.

Hyperspectral imaging technology [9] uses an imaging spectrometer with a spectral range from ultraviolet to near infrared (200–2500 nm). Tens or hundreds of spectral bands are continuously imaged within the spectral coverage of the target object. While acquiring the spatial feature image of the object, it also acquires the spectral information of the measured object [10]. It includes comprehensive technologies including precision optical technology, detector technology, mechanics, computer technology, signal detection technology, and information analysis technology knowledge. Its application area is shown in Figure 1.

Hyperspectral imaging technology can not only obtain the spatial information of the object but also obtain the spectral information of the object. Hyperspectral imaging technology can obtain continuous spectral bands of objects [11], and the number of bands reaches hundreds. Each pixel of the collected hyperspectral image has a complete reflectance spectrum curve. Compared with multispectral imaging technology, hyperspectral imaging technology can obtain more information in a narrower frequency band, and the spectral resolution can be accurate to a few nanometers.

#### 2.1.1. Nondestructive Testing Technology

**Figure 2:**
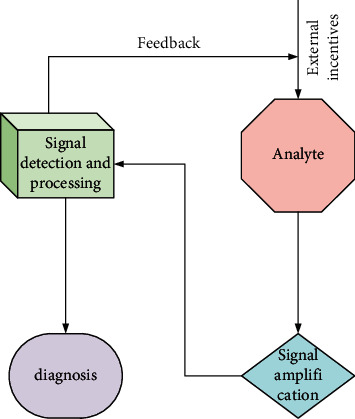
Basic measurement principle of nondestructive testing technology.

Nondestructive testing technology [12] is a comprehensive application discipline developed with modern physics, material science, electronic science, and computer science. According to different measurement principles and information processing methods, there are more than 70 types of nondestructive testing, covering all branches of modern physics. The basic measurement principle is shown in Figure 2. According to the different responses of the test object to external stimulation, the output information of the test object is collected, the correlation relationship with the input information is established, and then the physical and chemical properties of the test object can be diagnosed.

#### 2.1.2. Basic Principles of Spectroscopy

**Figure 3:**
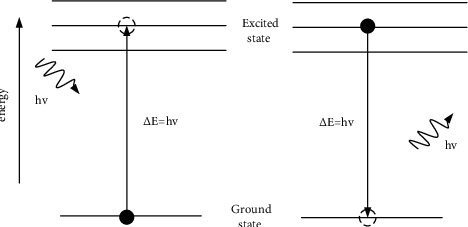
Schematic diagram of particle absorption or dissipation of energy.

**Figure 4:**
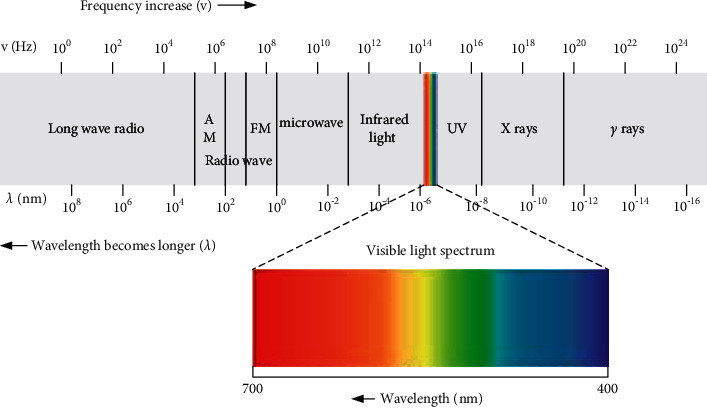
Spectral images of different ranges and the molecular motion modes generated by energy.

The matter is always in motion, and the atoms and molecules that makeup matter are also in motion. The rotation of electrons in molecules around atoms is called electronic movement. The vibration of atoms in a molecule is called molecular vibration. The rotation of the molecule itself is called molecular rotation [13]. Different exercise methods have different energies, divided by energy level, that is, electronic energy level, vibration energy level, and rotational energy level. When a substance is stimulated by a specific external energy, the state of molecular motion will change, and the energy level will also change accordingly. This form of change is achieved through the absorption or divergence of energy photons, as shown in Figure 3. Particles absorb energy photons and then transit from the ground state to the excited state, and from the excited state back to the ground state, and they emit energy photons. This form of energy is electromagnetic radiation, commonly known as light. Arranging electromagnetic radiation according to a certain energy level or frequency order is the electromagnetic radiation spectrum or spectrum [14]. As shown in Figure 4, according to the difference between absorption and divergence energy, it is divided into absorption spectrum and divergence spectrum.

Near-infrared spectroscopy [11] (780–2526 nm) is the earliest nonvisible spectrum discovered by mankind, and it is also one of the earliest researched spectra. The generation of near-infrared light is mainly caused by the internal vibration of polyatomic molecules. It contains a wealth of chemical bond information, such as hydrogen bond, bond strength, chemical species, and dielectric properties.

### 2.2. Trace Inspection

**Figure 5:**
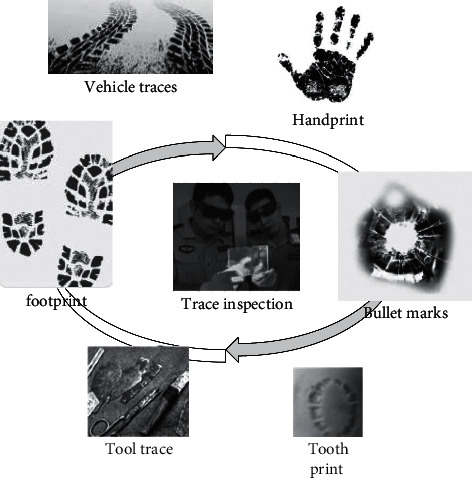
Main methods of trace inspection.

Trace inspection [15] is the analysis, identification, and judgment of various characteristics of the formation and change characteristics of criminal traces and the object of trace creation [16]. Therefore, in the process of trace inspection, the collection of traces is very important. The main method is shown in Figure 5.

In the process of trace inspection, any on-site traces need to be used as evidence and should be extracted from the site and preserved in their original form for use by inspectors. The main methods of extracting traces are as follows: transfer method, such as transferring trace materials to a specific carrier by a specific method; molding methods, such as using silicone rubber or plaster liquid to make trace models for three-dimensional traces; photocopying methods, such as the use of electrostatic adsorption to extract traces of dust; the original extraction method, such as direct collection and extraction of small-volume trace objects and trace-bearing objects. The photographic method is a method that can be used for all kinds of trace extraction in the process of trace collection. It can not only achieve lossless acquisition [17] but also perform multiple acquisition processes. Moreover, the photographic method is also a work step that must be carried out before trace extraction, and it is a necessary means to ensure the originality of traces.

The collection of traces includes the collection of pictures and text. It is a highly professional work. Only comprehensive and correct collection can reflect the actual work situation of on-site investigation and trace inspection, and the basic role of traces can be brought into play.

Image [18] has been used in forensics since it was invented in 1839. After entering the digital age, the frequency of use of images has become higher, an alternative to analog images—digital images have the advantages of environmental protection, low collection error rate, and convenient use, and it plays an increasingly important role in the process of trace inspection.

In the digitization of trace inspection, the collection of digital images is one of the important methods of trace collection. It mainly refers to the process of receiving light waves reflected or emitted by objects in the scene, recording and storing them, or obtaining image data through other scientific and technological equipment.

There are many ways to collect images. At present, there are four most commonly used collection methods in this article: using a digital camera to take photos directly, using a scanner to scan analog images, capturing video frames, and creating using drawing software. In the trace inspection, the collection of traces must be a true and objective reflection, so the image collection of traces mainly uses the first three methods.

#### 2.2.1. Tire Tracks on the Road

**Figure 6:**
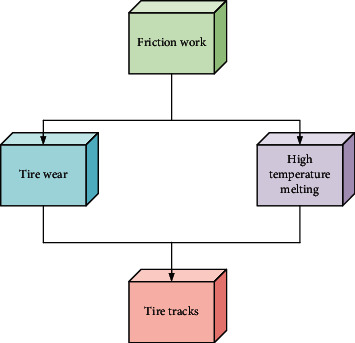
The formation mechanism of tire marks.

As the main component of the vehicle, tires play a role in supporting the weight of the vehicle, changing the direction of travel, transmitting braking and driving force, and alleviating road impact. Carbon black, which is a compounding agent for tire rubber materials, adheres to the road surface and leaves marks. In the case of high friction between the mud and the road, the heat release rate is lower than that of the mud of the tire, and the melting point of the tire and the road is relatively low, so the mud rubber becomes soft, black, peeling, and black when attached to the road. The tire traces on the road, such traces can be extracted from the debris of mud rubber [19], and the principle is shown in Figure 6.

In the process of collecting tire traces at the accident site, the tire traces of vehicles related to the accident can be judged according to the new degree of tire traces. The tire marks of a puncture on the road are flat sandwich marks with dust marks and rubber particle marks. Dust traces are usually only displayed on newly repaired roads. In most cases, those are potential traces. In contrast, the rubber particles of cement are easier to observe on the road track (usually displayed when the mud and the road slide against each other). The new tire truck leaves a lot of rubber particles on the road, and the color becomes darker. If the tire of the vehicle involved in the accident slips, then there will be obvious scars on the road surface. Old tire trucks generally have no particles of tire marks. Generally speaking, the new tire tracks are the tire tracks of the vehicles involved in the accident.

#### 2.2.2. On-Site Shoe Sole Trace Pattern Image Extraction Method

There are various methods for extracting pattern images of shoe sole traces on-site [20], but the determination of the extraction method is mainly based on various conditions such as the trace-bearing object of sole traces and the complexity of the site.

The photographic extraction method is the most frequently used method of fixing and extracting on-site traces without damage. Physical evidence photography is mainly used to take photos of shoe sole traces at the scene. This is also an effective way to faithfully record the original state, location, and surrounding relationships of the sole trace pattern image. However, the use of photography to extract images of shoe sole traces will also encounter some problems. If the scale bar and the sole trace surface are not in the same plane, and the height difference is still relatively large, then this will cause the measurement error to become larger due to the perspective deformation of the near and far. The requirements for photographing conditions are relatively strict, so when using photographing methods to extract the image of shoe sole trace patterns, it must be strictly in accordance with the specifications.

The photocopy extraction method is mainly aimed at flat footprints, especially flat dust footprints. The photocopy extraction method transfers the sole traces from the trace-bearing object to other objects with larger color contrast, which is convenient for observation and photo fixation. Copy extraction methods include the electrostatic copy method and paste copy method. Among them, the electrostatic photocopying method is mainly to add or subtract layers of dust sole traces left on the surface of relatively flat and dry objects such as cement floors, terrazzo floors, wooden surfaces, floors, towels, and textiles. The paste copying method is to use relatively a wide palm print tape or sole trace special tape to attach clear dust flat sole traces or sole traces after powder brushing and stained fixed sole traces, etc.

The mold extraction method is mainly aimed at the three-dimensional sole traces. After taking photos of the three-dimensional sole traces, a model of the sole traces must be made. Molding methods include the plaster molding method and cassia gum molding method. Molding methods are also different for different mark-bearing objects. For example, mold making on snow cannot use water at room temperature to prepare plaster liquid. It is necessary to use water temperature close to snow to mold, or use snow trace wax, snow sole trace fixative, etc. to fix the snow sole traces before molding. The cost of cassia glue molding is higher, but in major cases, the cassia glue molding method can be used for local small sole traces. The model made of cassia glue is elastic, not easy to break or break, and the shape is delicate, which can better reflect the characteristics of the traces of the sole.

For the visualization of potential footprints at the crime scene, powder and chemical visualization methods are often used. The chemical display method also includes the red blood salt color method of dust sole traces, the value indicator method of dust sole traces, the “502” glue display method, and the tetramethylaniline solution method of blood footprints.

No matter which extraction method is used, the extraction must be performed in accordance with the specifications; otherwise, the extracted sole trace pattern image will lack information and affect the subsequent processing work.

#### 2.2.3. Footprint Detection Research Technology

*(1) Height Identification*. Most surveys and statistics show that there are many types, many patterns, and high overlapping characteristics of footprints left on crime scenes. To a certain extent, it affects the reflection of traditional footprint features, such as the interaction between legs and bearing objects during walking. At the same time, the extraction method of on-site footprints is also one of the important factors affecting the inspection results [21]. First of all, in order to improve the accuracy of the inspection, before analyzing and inspecting the footprints, it is necessary to determine the symptom extraction method to be used based on various footprints and various on-site environments.

*(2) Age Analysis*. In the past, in some films and TV works, it was often seen that the police judged the age of the characters left by the footprints left at the crime scene. Judging their age through footprints is very helpful in investigating criminal cases.

Age is the length of time from birth to death, usually expressed in “years.” Age is not only a natural sign based on biology but also an important time sign in physiological processes. The characteristics of human footwork are also closely related to age. The characteristics of footwork reflect a person's physiological state and can correctly reflect person's various physiological states.

### 2.3. Image Denoising

At present, many methods have been proposed to filter out the noise in the image. The smoothing of images is generally divided into two categories: global processing and local processing. Global processing is to correct the entire image or most of the image. This method is relatively computationally expensive. Local processing is the use of local operators on the image. Calculating the neighborhood of a specific pixel greatly reduces the amount of calculation.

The following sections describe several commonly used image denoising methods:

#### 2.3.1. Neighborhood Average Method

The neighborhood average method [22], called the average filtering method, is a simple spatial region processing method. The basic idea is to replace the gray value of each pixel with the average value of the gray values of several pixels. *f*(*a*, *b*) is a noisy specific *K* × *K* image. After the neighborhood averaging process, the image *h*(*a*, *b*) is obtained. *h*(*a*, *b*) is determined by the following formula:

(1) $$\begin{array}{c} h\left(a,b\right)=\frac{1}{j}\underset{\left(i,j\right)\in D}{\sum }f\left(i,j\right). \end{array}$$

#### 2.3.2. Median Filtering Method

The median filter method [23] is the most widely used statistical filter in image processing, and it is also the most famous sequential statistical filter. The neighborhood averaging method blurs the edges of the image while removing noise. In contrast, the middle finger filtering is better than neighborhood averaging.

Median filtering is done on the one-dimensional sequence *f*_1_, *f*_2_, *f*_3_,…, *f*_*k*_. The length of the window is taken as *j*, and the number of *j* from the sequence *f*_*x*−*v*_,…, *f*_*x*−1_, *f*_*x*_, *f*_*x*+1_,…, *f*_*x*+*v*_ is extracted as the center point of the window, where

(2) $$\begin{array}{c} v=\frac{\left(j-1\right)}{2}. \end{array}$$

The number of *j* is arranged by size at a time, and the middle value is taken as the output value:

(3) $$\begin{array}{c} b_{x}=\text{med}\left\{f_{x-v},\ldots ,f_{x-1},f_{x},f_{x+1},\ldots ,f_{x+v}\right\}x\in k. \end{array}$$

The two-dimensional median filter is represented by the following formula:

(4) $$\begin{array}{c} b_{x}=\text{med}\left\{f_{xy}\right\}. \end{array}$$

#### 2.3.3. Low-Pass Filtering Method

Both the neighborhood averaging method and the median filtering method process the image in the spatial domain. The low-pass filtering method [24] is a method of processing images in the frequency domain.

The mathematical expression of the filter is as follows:

(5) $$\begin{array}{c} H\left(a,b\right)=G\left(a,b\right)F\left(a,b\right). \end{array}$$

Among them, *F*(*a*, *b*) is the Fourier transform of the original image, *H*(*a*, *b*) is the Fourier transform of the image smoothed by the filter, and *G*(*a*, *b*) is the transfer function of the filter.

The edges, jumps, and grain noise of the image are the high-frequency components of the image. The background area represents the low-frequency components, so the simplest low-frequency filter is to obtain the high-frequency components in the Fourier transform of the image. The corresponding filter is called a two-dimensional ideal low-pass filter (ILPF), and its transfer function is as follows:

(6) $$\begin{array}{c} G\left(a,b\right)=\left\{\begin{array}{cc} 1, & S\left(a,b\right)\leq S_{0},\\ 0, & S\left(a,b\right)>S_{0}. \end{array}\right. \end{array}$$

Among them, *S*_0_ is the designated non-negative value, and *S*(*a*, *b*) is the distance between point (*a*, *b*) and the origin of the frequency rectangle. In addition to ideal low-pass filters, low-pass filters also include Butterworth low-pass filters (BLPFs) and Gaussian low-pass filters (GLPFs). This article uses the Gaussian slow path filter. The transfer function of the Gaussian slow path filter is as follows:

(7) $$\begin{array}{c} G\left(a,b\right)=q^{-S^{2}\left(a,b\right)/2S_{0}^{2}}. \end{array}$$

Among them, *S*(*a*, *b*) is the distance from the origin of the Fourier transform.

There are many methods for image sharpening, such as high-pass filtering, gradient sharpening, and Laplacian sharpening. The high-pass filter includes ideal high-pass filter (IHPF), Butterworth high-pass filter (BHPF), and Gaussian high-pass filter (GHPF). This article only introduces gradient sharpness and Laplacian sharpening.

*(1) Gradient Sharpening*. Gradient processing is often used in industrial inspections, auxiliary manual defects, or more general automatic inspection preprocessing. For image *f*, the gradient at point (*a*, *b*) is as follows:

(8) $$\begin{array}{c} H\left[f\left(a,b\right)\right]={\left[{\left(\frac{\gamma f}{\gamma a}\right)}^{2}+{\left(\frac{\gamma f}{\gamma a}\right)}^{2}\right]}^{1/2}. \end{array}$$

For discrete images, the above formula can be approximated by the difference method to obtain the following equation:

(9) $$\begin{array}{c} H\left[f\left(a,b\right)\right]={\left\{{\left[f\left(a,b\right)-f\left(a-1,b\right)\right]}^{2}+{\left[f\left(a,b\right)-f\left(a,b-1\right)\right]}^{2}\right\}}^{1/2}. \end{array}$$

In order to facilitate programming and improve calculations, it can be further simplified as follows:

(10) $$\begin{array}{c} H\left[f\left(a,b\right)\right]=\left|f\left(a,b\right)-f\left(a-1,b\right)\right|+\left|f\left(a,b\right)-f\left(a,b-1\right)\right|. \end{array}$$

*(2) Laplacian Sharpening*. Like the gradient, Laplace operation is also a linear combination of partial differential operations, which is a linear operation accompanied by rotation invariance as follows:

(11) $$\begin{array}{c} \nabla^{2}f=\frac{\gamma^{2}f}{\gamma^{2}a}+\frac{\gamma^{2}f}{\gamma^{2}a}. \end{array}$$

For discrete digital images, the Laplacian operator can be expressed as follows:

(12) $$\begin{array}{c} \nabla^{2}f=\frac{\gamma^{2}f}{\gamma^{2}a}+\frac{\gamma^{2}f}{\gamma^{2}a}=f\left(a+1,b\right)+f\left(a-1,b\right)+f\left(a,b+1\right)+f\left(a,b-1\right)-4f\left(a,b\right). \end{array}$$

The following formula is used to deal with the image blur caused by the diffusion effect:

(13) $$\begin{array}{c} h\left(a,b\right)=f\left(a,b\right)-k\theta \nabla^{2}f\left(a,b\right). \end{array}$$

Among them, *kθ* represents the coefficient related to the diffusion effect. The value of *kθ* must be moderate; otherwise, it will affect the sharpening effect of the image. If *kθ*=1 is taken, then the formula is transformed as follows:

(14) $$\begin{array}{c} h\left(a,b\right)=5f\left(a,b\right)-f\left(a+1,b\right)-f\left(a-1,b\right)-f\left(a,b+1\right)-f\left(a,b-1\right). \end{array}$$

### 2.4. Least Squares Support Vector Machine

There is a modeling set {*a*_*n*_, *b*_*n*_}_*n*=1_^*K*^ composed of *K* data, where the input data are *a*_*n*_ ∈ *R*^*K*^ and the output data are *b*_*n*_ ∈ *R*. Using a nonlinear function *γ*(·), the input data are mapped to a high-dimensional feature space and a relationship model is established:

(15) $$\begin{array}{c} b\left(a\right)=w^{S}\gamma \left(a\right)+y. \end{array}$$

In the formula, *w* ∈ *R*^*k*^ is the weight vector and *b* is the bias value. When using least squares support vector machine to solve, the function fitting problem can be described as the following optimization problem:

(16) $$\begin{array}{c} \min M\left(w,q\right)=\frac{1}{2}w^{S}w+\frac{1}{2}\mu \overset{K}{\underset{n=1}{\sum }}q_{n}^{2}. \end{array}$$

The constraints are as follows:

(17) $$\begin{array}{c} b_{n}=w^{S}\gamma \left(a\right)+y+q_{n},n=1,\ldots ,K. \end{array}$$

In the formula, *R*^*k*^⟶*R*^*k*_*n*_^ is the kernel space mapping function, *μ* is the penalty coefficient, and *q*_*n*_ is the error variable. According to the formula, the model is transformed into the dual space to solve it, and the following Lagrange function is obtained:

(18) $$\begin{array}{c} L\left(w,y,q,x\right)=M\left(w,q\right)-\overset{K}{\underset{n=1}{\sum }}x_{n}\left\{w^{S}\gamma \left(a_{n}\right)+y+q_{n}-b_{n}\right\}. \end{array}$$

In the formula, the Lagrange multiplier *x*_*n*_ ∈ *R* is called the support value. The partial derivative of each variable is obtained to get the following conditional equation:

(19) $$\begin{array}{c} \frac{\phi L}{\phi w}=0⟶w=\overset{K}{\underset{n=1}{\sum }}x_{n}\gamma \left(a_{n}\right),\\ \frac{\phi L}{\phi y}=0⟶\overset{K}{\underset{n=1}{\sum }}x_{n}=0,\\ \frac{\phi L}{\phi q_{n}}=0⟶x_{n}=\mu q_{n},n=1,\ldots ,K,\\ \frac{\phi L}{\phi x_{n}}=0⟶w^{S}\gamma \left(a_{n}\right)+y+q_{n}-b_{n}=0,n=1,\ldots ,K. \end{array}$$

After eliminating the variables *w* and *q*, the system of linear equations can be obtained:

(20) $$\begin{array}{c} \left[\begin{array}{cc} 0 & {\overset{⟶}{1}}^{S}\\ \overset{⟶}{1} & \rho +\mu^{-1}I \end{array}\right]\left[\begin{array}{c} y\\ x \end{array}\right]=\left[\begin{array}{c} 0\\ b \end{array}\right]. \end{array}$$

In the formula, $\left\{\begin{array}{c} \begin{array}{c} b=\left[b_{1},\ldots ,b_{K}\right],\\ \overset{⟶}{1}=\left[1,\ldots ,1\right],\\ x=\left[x_{1},\ldots ,x_{K}\right], \end{array}\\ \rho =\left\{\rho_{nl}|n,l=1,\ldots ,K\right\}, \end{array}\right.$ and *ρ*_*nm*_=*γ*(*a*_*n*_)^*S*^*γ*(*a*_1_)=*N*(*x*_*k*_, *x*_*l*_), *n*, *l*=1,…, *K*.

*N*(*x*_*k*_, *x*_*l*_) is the kernel function that must satisfy Massa's theorem. The commonly used kernel functions include linear kernel functions, polynomial kernel functions, radial basis kernel functions, and multilayer acceptor kernel functions. The kernel function used in this article is a nonlinear radial basis kernel function:

(21) $$\begin{array}{c} N\left(x_{k},x_{l}\right)=\exp\frac{-{\left(a-b\right)}^{2}}{2\varepsilon^{2}}. \end{array}$$

Thus, the fitting model of the least squares support vector machine can be obtained as follows:

(22) $$\begin{array}{c} b\left(a\right)=\overset{K}{\underset{n=1}{\sum }}x_{n}N\left(a,a_{n}\right)+y. \end{array}$$

## 3. Test Experiment of Trace Inspection System Based on Hyperspectral Imaging Technology

### 3.1. Retrieval Performance Experiment of Trace Inspection System

In order to verify the retrieval performance of the imaging spectrum image trace inspection system proposed in this study, the average retrieval accuracy before and after feature randomization encryption is compared in the experiment. It also compares the average precision and retrieval time of the encryption method in this study and the order-preserving encryption method in image retrieval. It is a comparison of the retrieval performance of the imaging spectral image security retrieval system before and after the introduction of relevant feedback.

**Figure 7:**
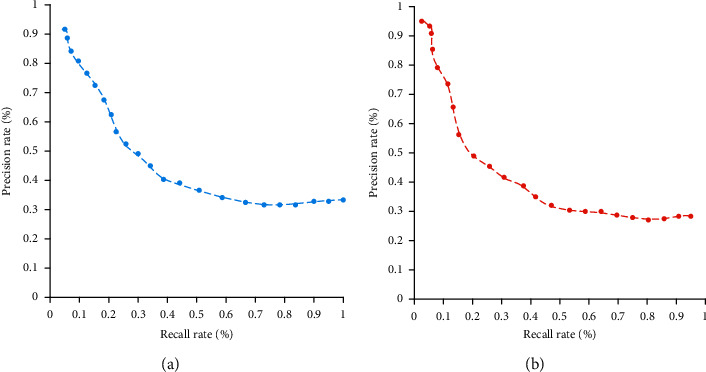
Recall rate-precision rate curve before and after feature encryption. (a) Before feature randomization and encryption. (b) After feature randomization and encryption.

In order to prove the effectiveness of the feature randomization encryption method, this experiment compares the recall curve and average accuracy of image retrieval before and after encryption.  Figure 7 shows the recall-precision ratio curve before and after feature encryption. It can be seen from the figure that the precision and recall rates after feature encryption have not changed much compared with that before feature encryption.

In order to visually see the impact of feature encryption on the retrieval accuracy, Table 1 lists the results of the average retrieval accuracy before and after encryption.

It can be seen from the table that the average precision before encryption using feature randomization is 86.10% and after encryption is 85.25%, and the overall performance of the image retrieval average precision before and after encryption is equivalent. That is, the method in this study has little effect on the accuracy of the retrieval system. The method in this study can effectively protect the image feature information while ensuring the accurate retrieval of imaging spectral images.

**Figure 8:**
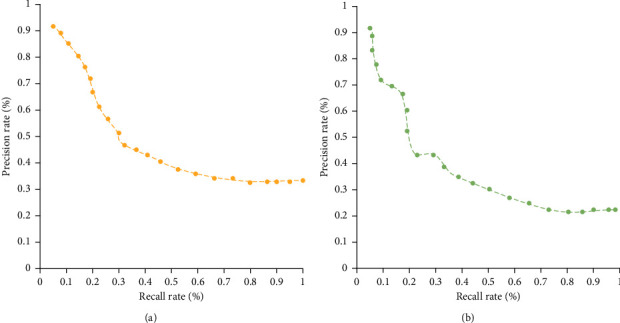
Recall rate-precision rate curve of two different feature encryption methods. (a) Characteristic randomization encryption. (b) Order-preserving encryption.

In order to further prove the effectiveness of the feature randomization encryption method in this study, this experiment compares the exact recall rate and the average precision rate with order-preserving encryption.  Figure 8 shows the recall-precision curve of the two encryption methods. It can be seen from the figure that the retrieval performance of the feature randomization encryption method in this study is better than that of the existing order-preserving encryption methods in the laboratory.

In order to compare the retrieval performance of the two methods intuitively, the average precision of image retrieval of the two methods is given in Table 2.

It can be seen from the table that the average precision of the randomized encryption method in this study is 85.25%, and the average precision of the order-preserving encryption method is 83.26%, which is about 2% higher. It proves the effectiveness of this method. The table also lists the comparison of encryption time and retrieval time of two different feature encryption methods. The feature encryption time of the method in this study is 5.0 × 10 − 3 s, the time to retrieve an image after encryption is 1.0 s, the order-preserving encryption method requires 1.10 s for feature encryption, and the time to retrieve an image after encryption is 3.0 s. Whether in terms of feature encryption or retrieval speed, the performance of this method is optimal. The main reason is that the form of the hash code of the depth spectrum-spatial feature in this article speeds up the calculation speed.

### 3.2. Hyperspectral Image Denoising Experiment

If there is a large amount of hyperspectral image data, then it will affect the subsequent processing. Therefore, the ENVI software is used to cut a part of the calibration image without data and delete redundant data information. Since hyperspectral technology was originally used in the field of remote sensing, it was originally used to process hyperspectral remote sensing images.

The amount of data in hyperspectral images is very large, the number of bands is large, and the data and information between bands are repetitive and redundant. The original CCD detection that records the reflection spectrum of each band is very sensitive, and the existence of dark current has an impact on the experimental data. The steps to reduce hyperspectral image noise are as follows: open the ENVI software, select the hyperspectral image after the black and white frame calibration, click [MNF Rotation] under the [Conversion] function key, select [Forward MNF], and perform the positive change with minimum noise.

**Figure 9:**
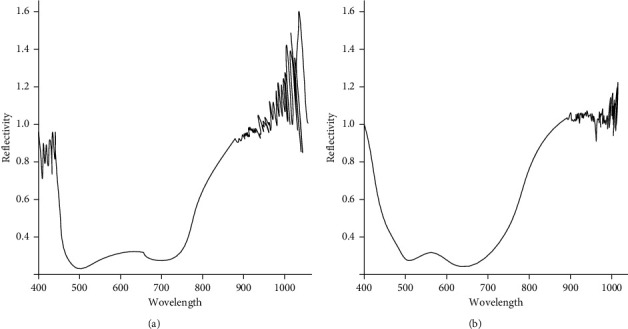
The difference of the reflectance spectrum curve of the hyperspectral image before and after noise reduction. (a) Reflectance spectrum curve of original hyperspectral image. (b) Reflectance spectrum curve of hyperspectral image after noise reduction.

The spatial subset adjusts the size of the sensing area of the hyperspectral image, so that the tracking range to be processed is included in the sensing area, and redundant background information is suppressed to a minimum. In the second step, the inverse transformation of the MNF rotation algorithm is performed. The noise-free file processed in the previous step is selected, the image size is set to be the same as the size of the sensing area in the previous step, and the noise-reduced file and the noise-removed hyperspectral image are saved, as shown in Figure 9.

It can be seen from the figure that after the noise reduction of the hyperspectral image, the burrs of the reflection spectrum curve of the traces are reduced and become smoother.

### 3.3. Hyperspectral Image Fusion of Different Bloodstain Samples

Because the pictures obtained in this experiment are hyperspectral images of all bands, not all of them are suitable for image fusion processing. This article needs to find images that are valuable for the experiment to be fused. As far as blood handwriting pictures are concerned, there are two factors that affect the fusion effect: one is the clarity of the blood handwriting itself and the other is the clarity of the background image of the blood handwriting carrier. Generally speaking, blood writing and carrier background under different wavebands will not reach the clearest degree at the same time, because the degree of absorption and response to the same wavelength of light is different between the blood and the carrier. Therefore, this article needs to select two types of pictures for fusion: one is the picture with the clearest blood writing and the clearness of the carrier background. The second is the clearest background image of the carrier, and the clearness of blood handwriting is uncertain. Combining the two pictures together can get a clear picture of the blood handwriting image and the carrier background image. The experimental results are listed in Table 3.

Through the evaluation indicators in the table, it can be found that after the fusion, the average value of the blood handwriting image increases, and the visual effect of naked-eye observation is improved. The standard deviation of blood handwriting images increases, and the amount of information tends to maximize. The average gradient of the blood handwriting image increases, and its sharpness increases. The information entropy increases, and the amount of information increases. It shows that the image obtained by wavelet fusion is clearer and more informative than the blood handwriting image of a single band, which can greatly improve the ability of hyperspectral bloodstain detection.

Regarding the hyperspectral image fusion of blood fingerprint samples, 6 images that meet the requirements were selected for fusion in the experiment. The selected images and the fusion results are listed in Table 4.

Through the evaluation indicators in the table, it can be found that after the fusion, the average value of the blood fingerprint image increases, and the visual effect of naked-eye observation is improved. The standard deviation of blood fingerprint images increases, and the amount of information tends to maximize. The average gradient of the blood fingerprint image increases, and its sharpness increases. The information entropy increases, and the amount of information increases. It shows that the image obtained by wavelet fusion is clearer than a single-band blood fingerprint image and has a larger amount of information, which can greatly improve the ability of hyperspectral bloodstain detection.

For the hyperspectral image fusion of conventional bloodstain samples, three images that meet the requirements are selected for fusion processing in the experiment. The selected images are listed in Table 5.

Through the evaluation indicators in the table, it can be found that after fusion, the average value of conventional bloodstain images increases, and the visual effect of naked-eye observation is improved. The standard deviation of conventional bloodstain images increases, and the amount of information tends to maximize. The average gradient of the conventional bloodstain image increases, and its sharpness increases. The information entropy increases, and the amount of information increases. It shows that the image obtained by wavelet fusion is clearer and more informative than the conventional bloodstain image of a single band, which can greatly improve the ability of hyperspectral bloodstain detection.

## 4. The Image Trace Inspection Capability of the Trace Inspection System on Account of Hyperspectral Imaging Technology

**Figure 10:**
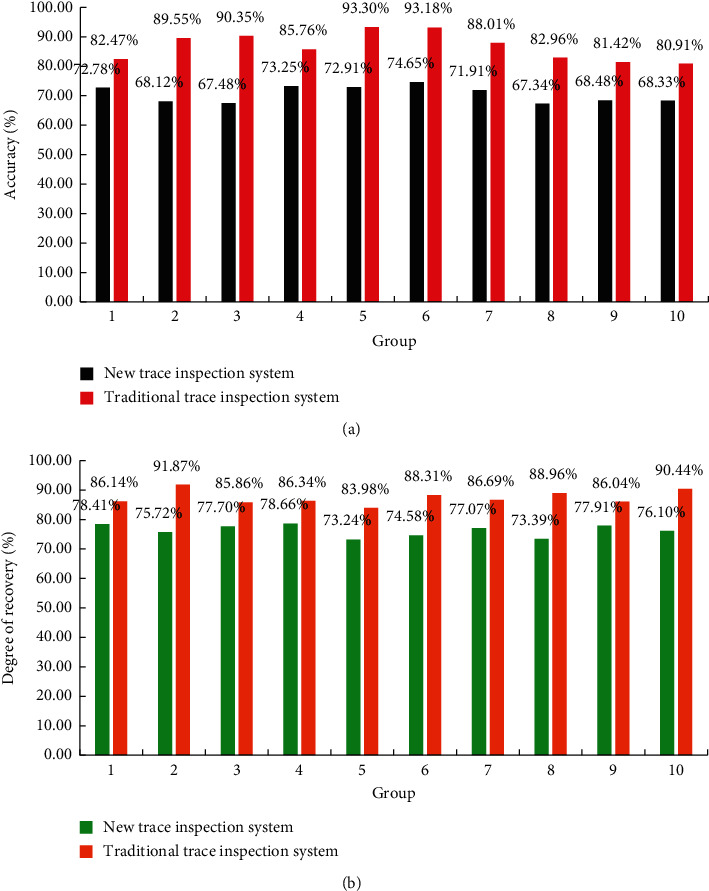
Comparison result of trace inspection system based on hyperspectral imaging technology and traditional trace inspection system. (a) Accuracy comparison. (b) Comparison of recovery.

Based on the hyperspectral imaging technology and the knowledge of trace inspection, this study designs a trace inspection system based on hyperspectral imaging technology. The system can improve the degree of trace recovery and also improve the accuracy of the image. The system can inspect residual traces such as car tires, shoe soles, and blood stains. In this regard, this study designs a set of controlled experiments. They were compared with a trace inspection system based on hyperspectral imaging technology and a traditional trace inspection system. There are 10 sets of pictures in each group, and the detection accuracy and recovery degree are compared respectively. The experimental results are shown in Figure 10.

It can be seen from the figure that the accuracy of the trace inspection system based on hyperspectral imaging technology can reach 80%–95%, while the traditional one is only 67%–75%. Therefore, the trace inspection system of hyperspectral imaging technology designed in this study can improve the accuracy by 5%–28%. The recovery degree of the trace inspection system of hyperspectral imaging technology can reach 80%–92%, while the traditional one is only 73%–79%. It shows that the trace inspection system of hyperspectral imaging technology can be improved by 1%–19%, compared with the traditional one. By analyzing the experimental results, the trace inspection system based on hyperspectral imaging technology has the characteristics of high accuracy and high image recovery ability compared with the traditional trace inspection system. This is very important for criminal investigation trace inspection.

## 5. Conclusion

This study mainly studies the application of hyperspectral imaging technology in trace inspection. In order to explore how it can be used in trace inspection, this article combines the nondestructive testing technology of hyperspectral imaging technology. By recognizing images of residual traces such as car tires, shoe soles, and blood stains, and then combining image denoising and least squares support vector machine methods, this article denoises and restores the images. This study also designs the retrieval performance experiment of the trace inspection system to verify the performance problems of the hyperspectral imaging technology. It designed a hyperspectral image noise reduction experiment to verify its degree of noise reduction. It also designed a hyperspectral image fusion experiment of blood stain samples to verify the fusion recovery capability of hyperspectral imaging technology.

## Figures and Tables

**Table 1:** Comparison of average precision before and after feature encryption.

Type	Characteristic encryption time	Retrieve time after encryption	Traditional method

Feature randomization encryption	5.0*∗*10 − 3 s	1.10 s	2.13 s
Order-preserving encryption	1.10 s	3.0 s	5.12 s
Average precision rate (%)	86.10	85.25	—

**Table 2:** Comparison of average precision of two different feature encryption methods.

Type	Characteristic encryption time	Retrieve time after encryption	Traditional method

Feature randomization encryption	5.0*∗*10 − 3 s	1.10 s	2.13 s
Order-preserving encryption	1.10 s	3.0 s	5.12 s
Average precision rate (%)	85.25	83.26	—

**Table 3:** Related evaluation indexes of blood handwriting wavelet fusion image.

Image	Information entropy	Average gradient	Average value	Standard deviation

A	4.26	5.55	26.43	28.94
B	4.82	6.23	30.34	29.65
C	4.51	6.13	25.76	33.21
D	5.35	5.82	27.43	30.84
E	4.36	5.31	29.43	27.65
F	5.21	6.33	32.84	25.64
G	5.56	5.71	23.45	29.34
H	5.04	5.16	28.64	31.54
I	6.21	10.26	55.27	47.68

**Table 4:** Related evaluation indexes of blood fingerprint wavelet fusion image.

Image	Information entropy	Average gradient	Average value	Standard deviation

A	4.35	5.31	26.41	29.46
B	5.31	6.71	30.54	28.64
C	4.67	6.13	26.64	31.56
D	6.13	6.81	28.64	31.64
E	6.23	6.12	29.64	32.91
F	5.12	8.61	25.31	26.94

**Table 5:** Related evaluation indexes of conventional bloodstain wavelet fusion image.

Image	Information entropy	Average gradient	Average value	Standard deviation

A	4.58	5.39	30.77	22.51
B	4.21	5.67	31.23	25.39
C	5.33	10.37	43.46	40.23

## Data Availability

The experimental data used to support the findings of this study are available from the corresponding author upon request.
